# Chromothripsis 18 in multiple myeloma patient with rapid extramedullary relapse

**DOI:** 10.1186/s13039-018-0357-5

**Published:** 2018-01-18

**Authors:** Jan Smetana, Jan Oppelt, Martin Štork, Luděk Pour, Petr Kuglík

**Affiliations:** 1 Laboratory of Molecular Cytogenetics, Institute of Experimental Biology, Faculty of Science, Masaryk University, Kotlářská 2, 602 00 Brno, Czech Republic; 2 Department of Medical Genetics, University Hospital, Brno, Czech Republic, Černopolní 9, Brno, Czech Republic; 3 CEITEC-Central European Institute of Technology, Masaryk University, Kamenice 5, 625 00 Brno, Czech Republic; 4 National Centre for Biomolecular Research, Faculty of Science, Masaryk University, Kotlářská 2, 611 37 Brno, Czech Republic; 5 Department of Internal Medicine-Hematooncology, University Hospital Brno, Jihlavská 20, 62500 Brno, Czech Republic

**Keywords:** Multiple myeloma, Chromothripsis, Array-CGH, NGS, Mutation screening

## Abstract

**Background:**

Catastrophic chromosomal event known as chromothripsis was proven to be a significant hallmark of poor prognosis in several cancer diseases. While this phenomenon is very rare in among multiple myeloma (MM) patients, its presence in karyotype is associated with very poor prognosis.

**Case presentation:**

In our case, we report a 62 year female patient with rapid progression of multiple myeloma (MM) into extramedullary disease and short overall survival (OS = 23 months). I-FISH investigation revealed presence of gain 1q21 and hyperdiploidy (+ 5,+ 9,+ 15) in 82% and 86%, respectively, while *IgH* rearrangements, del(17)(p13) and del(13)(q14) were evaluated as negative.

Whole-genome profiling using array-CGH showed complex genomic changes including hyperdiploidy (+ 3,+ 5,+ 9,+ 11, + 15,+ 19), monosomy X, structural gains (1q21-1q23.1, 1q32-1q44, 16p13.13-16p11.2) and losses (1q23.1-1q32.1; 8p23.3-8p11.21) of genetic material and chromothripsis in chromosome 18 with 6 breakpoint areas. Next-generation sequencing showed a total of 338 variants with 1.8% (6/338) of pathological mutations in *NRAS* (c.181C > A; p.Gln61Lys) or variants of unknown significance in *TP53, CUX1* and *POU4F1.*

**Conclusions:**

Our findings suggest that presence of chromothripsis should be considered as another important genetic hallmark of poor prognosis in MM patients and utilization of genome-wide screening techniques such as array-CGH and NGS improves the clinical diagnostics of the disease.

**Electronic supplementary material:**

The online version of this article (10.1186/s13039-018-0357-5) contains supplementary material, which is available to authorized users.

## Background

Multiple myeloma (MM) is characterized by malignant proliferation of clonal plasma cells (PCs) and accumulation of those cells in the bone marrow, formation of osteolytic lesions and presence of monoclonal immunoglobulin in serum and/or urine [1]. It is well established that development of MM is characterized by clinical and biological heterogeneity driven by proliferation of best adapted clone(s) of PCs, based on Darwin’s theory of evolution of species [2]. There are well-known correlations between prognosis and several chromosomal aberrations detected by interphase fluorescence in situ hybridization (I-FISH). Deletion of *TP53* in 17p13 loci, translocation t(4;14)(p16;q32) and gain(1)(q21) detected by I-FISH are considered as well established hallmarks of adverse prognosis for MM, both newly diagnosed and relapsed [3, 4]. However, implementation of genome-wide screening techniques, such as array-CGH or recently next generation sequencing (NGS), into clinical practice allows precise description of genetic heterogeneity of cancer diseases, including MM [5, 6]. In our previous study, we showed that array-CGH in combination with cell sorting is able to detect copy number alterations (CNAs) in 100% of MM and 65% of MGUS patients and could be used for monitoring of development of CNAs in clonal populations in MM patients [7, 8]. Other studies also showed that utilization of genome-wide screening techniques allows detection of specific types of genetic lesions with prognostic impact, such as homozygous deletions, loss of heterozygosity (LOH) or chromothripsis in MM [9–12].

The concept of genome chaos was has been recently established a results of extreme stress conditions to cells. Exposure to stress factors leads to complex genomic re-organization, which could ultimately give rise of stable genome, however with massive subtypes of chromosome rearrangements known as chromoanasynthesis, chromoanagenesis, chromoplexy or chromothripsis [13, 14]. Chromothripsis (CTH) has been established as a catastrophic event causing complex chromosomal rearrangements involving the shattering of a single chromosome, a small group of chromosomes, or a single chromosome arm [15]. The fragments, or a subset of the fragments, are then stitched together by non-homologous end joining, but in wrong order, place or orientation or also could be missing (copy number loss) or there could be extra copies (copy number gains of DNA) [16]. Due to nature of this phenomenon, it is not surprising that several recent studies showed that incidence of CTH is associated with extremely poor prognosis in cancer diseases as well as severe phenotype effects in congenital diseases [17–19]. In MM diagnosis, CTH is considered to be a rare chromosomal aberration with incidence of 1%; however, it is associated with a very short overall survival of newly diagnosed patients [20].

In this report, we would like to describe the case of a female MM patient with CTH affecting chromosome 18, hyperdiploid karyotype and pathological mutation in oncogene *NRAS* and impact on development of the disease. To our knowledge, this is the first published case with CTH 18 and complex genomic screening.

## Case presentation

Female patient without significant comorbidities was diagnosed in 01/2012 at the age of 62 years with symptomatic IgG MM, Durie-Salmon stage III. A, ISS stage 2. At the time of diagnosis, CRAB criteria (hypercalcemia - renal failure – anemia – bone lesions) were met by anemia and bone disease presented by pathological compression of C3 and L5 vertebrates as detected by MRI.

Treatment of the patient was initiated with 7 cycles of CVD (cyclophosphamid-velcade-dexamethasone). After induction, patient achieved partial remission (PR), and she subsequently underwent in 11/2012 autologous peripheral blood stem cell transplant (PBSCT) with melphalan (200 mg/m^2^) conditioning. After PBSCT treatment response did not improved and remained PR. Progression of disease occurred after 9 months with incidence of several extramedullary lesions in thoracic vertebrates Th5–7 (08/2013). The patient was then treated by RAD regimen (revlimid-adryamicin-dexamethasone) with supporting radiotherapy. After third cycle, no treatment response was achieved and patient’s condition was rapidly worsening. According to extremely aggressive clinical progression, recurrent severe infection complication and overall rapid worsening of the patient’s condition, we decided to withdraw oncological treatment and switch to symptomatic-palliative approach. Patient subsequently died due to progression of the disease in 11/2013. Overall survival of the patient was 23 months.

MACS (magnetic-activated cell sorting) technique was used at the time of diagnosis for obtaining enriched CD138^+^ PCs population for further genetic analyses. In our case, the population purity reached 91%. Detailed cell-sorting protocol was previously by us described elsewhere [21]. Expanded FISH panel for MM patients was used for detection of chromosomal abnormalities with known prognostic impact [22]. We found hyperdiploidy (+ 5,+ 9,+ 15) in 82% (82/100) and gain 1q21 (*CKS1B* loci) in 86% (86/100) of scored cells, while *IgH* rearrangement, del(17)(p13) and del(13)(q14) were evaluated as negative.

**Fig. 1:**
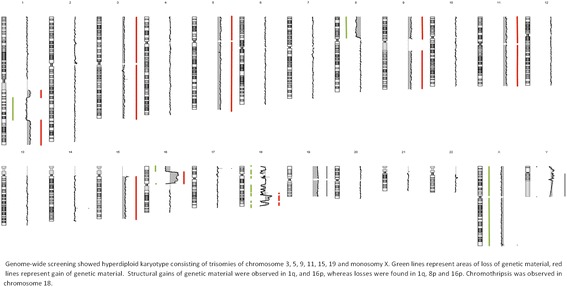
Overall graphical summary of CNAs in patients with hyperdiploid MM and CTH affected chromosome 18

**Fig. 2:**
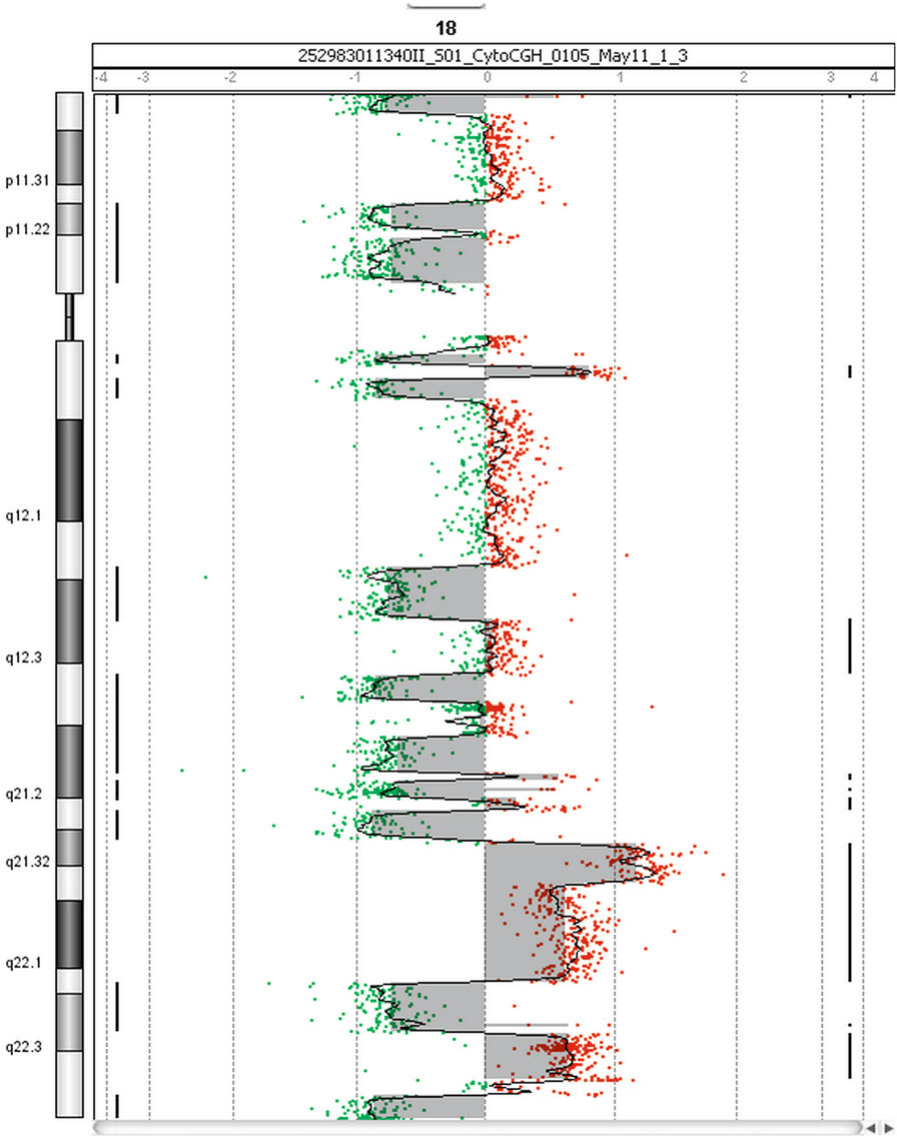
Detailed visualization of chromothripsis affecting of chromosome 18. Array-CGH detected 6 breakpoints, 12 areas of loss and 4 areas of gain of genetic material

Retrospective genome-wide screening showed overall 28 copy number aberrations (CNAs) with the use of microarray-based comparative genomic hybridization (array-CGH). Those aberrations include aneuploidies of odd-numbered chromosomes (+ 3,+ 5,+ 9, + 11, + 15,+ 19) and monosomy X. Structural CNAs were observed as deletions in 1q, 8p and 16p, while areas of gain of genetic material were found in 1q and 16q. Complex chromosomal changes were detected in chromosome 18 and positively identified as chromothripsis (Fig. 1). We observed 6 breakpoint areas, which were accompanied with 12 areas of heterozygous deletions (median of size 2.1 Mbp) and 4 areas of gain of genetic material (median of size 3.3 Mbp). Interestingly, we observed an area of 2.5 Mbp affected with amplification (5–6 copies) in 18q21 (Fig. 2). Moreover, in 16p13.13-16p11.2, we found another area of amplification approximately 22.5 Mb of size, harboring 259 genes spawning from *SOCS1* to *SLC6A10P*. Detailed overview on CNAs is shown in Additional file 1: Table S1.

**Fig. 3:**
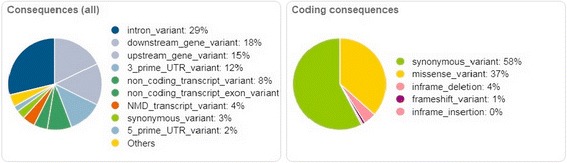
Overview of 338 variants from next-generation sequencing of 62 hematooncology associated genes from Sistema Genómicos custom panel

Mutation screening of 62 leukemia-related genes included in LMA-GeneSGKit® (Sistema Genómicos, Spain) showed total of 338 variants. Graphical overview of variant types is shown in Fig. 3. We identified 1.8% (6/338) potentially pathological variants or variants with unknown significance. The most importation mutation was found in *NRAS* as missense variant in exon 3 (c.181C > A; p.Gln61Lys). Variants with unknown significance were further observed in *RAF1, TP53, CUX1* and *POU4F1.* Detailed information about all variants is available in Additional file 2: Table S2.

## Discussion and conclusions

Identification of chromosomal aberrations in malignant PCs is one of the basic steps in MM staging, which has important impact on disease prognosis of MM disease [23]. FISH investigation with the help of immunostaining (cIg-FISH) or I-FISH performed on enriched CD138^+^ PCs population is still considered as golden standard for cytogenetic testing in MM patients. Metaphase cytogenetics is not suitable technique due to low mitotic activity of PCs, giving results in < 30% cases, while I-FISH in combination with cell sorting reaches 90% of positive investigations [24, 25].

Incidence of *IgH* translocations, loss of *TP53*, gain of genetic material in 1q21 area detected by FISH are considered to be negative prognostic factors for both newly diagnosed and relapsed patients [26–28]. While these markers are well established and FISH is considered as gold standard of genetic evaluations in MM, the advent of genome-wide screening techniques brought crucial novel information about biology of malignant PCs and genetic basis of the disease, ranging from evaluation of novel CNAs with prognostic impact to discovery of clonal heterogeneity as the principle of development of the disease, sequencing the whole genome of malignant PCs and findings of mutations in critical genes such as *BRAF*, which could be used for personalized treatment protocols [29, 30]. The utilization in genome-wide profiling in MM patients with the use of array-CGH technique showed that similarly to other hematological malignancies, malignant PCs can harbor a complex chromosomal aberration known as chromothripsis with incidence of 2% [31]. While the incidence of chromothripsis seems to be rare event in blood cancer diseases, it is only a portion of all types of genomic chaos, which is considered to be one the major contributors to cancer development and progression [32, 33]. The study of French Myeloma Group also showed that incidence of this catastrophic event detected by microarray techniques in genome of PCs is associated with very poor prognosis and aggressive course of the disease [20]. All these clinical features were present in our patient in a similar manner. This MM case represents a single occurrence of CTH (1.2%; 1/91) in our previously published array-CGH dataset [34]. Overall survival of this patient reached only 23 months, which is in good concordance with data from the French study (OS range from 6 to 32 months, relapse during 10 months from diagnosis). The incidence of chromothripsis affecting chromosome 18 was accompanied with presence of hyperdiploid karyotype, as it is not uncommon in cancer cells [35]. In addition, amplification in 18q affected several genes with known impact in hematological malignancies, such as *MALT1* (18q21.32), *BCL2* (18q21.33), and *KDSR* (18q21.33).

While using FISH investigation, when only gain 1q21 was detected as high-impact negative prognostic marker, retrospective utilization of array-CGH showed overall 28 CNAs with areas of amplification of DNA carrying loci of candidate oncogenes in 16p (*IL4R, IL21R*) and 18q21.32 (*PMAIP*1). Similar data were previously published with the use of microarray techniques in MM by other groups [36, 37]. Furthermore, losses in 1q and 8p were previously detected by genome-wide profiling in MM patients, however impact of those CNAs on prognosis remains unclear as they were part of complex genomic changes [38, 39].

Benchtop NGS approach together with custom panel of hematonocology-related genes showed possibly harmful variants in 1.8% (6/338). While pathological mutations in *NRAS* and *TP53* were previously described [40, 41], 3’UTR SNP variant:7,668,862 G > A in *TP53* was observed in a single patient with Li-Fraumeni syndrome, exon 7 indel CTGTT>C chr3:12,583,765 in *RAF1* was found in patient with Noonan syndrome according to Pubmed ICLS (Illumina-Clinical-Services-Laboratory) online public database (https://www.ncbi.nlm.nih.gov/clinvar/submitters/504895). The rest of the variants are annotated in public databases with unknown function, thus their impact is yet to be determined. Of those, intergenic missense variant in *CUX1 (*rs118010189) and G- > C chr13:78,602,199 mutation in *POU4F1* promoter flanking region were predicted as intolerant due to possible amino acid substitutions to and with effect of phenotype according to SIFT database [42].

According to aggressive phenotype of this aberration, we suggest to treat MM patients with CTH with the aim to reach the best available treatment response [31]. Triplet induction CVD followed by hi-dose chemotherapy and autoPBSCT was best available choice covered by rules of Czech health system at the time of diagnosis. Furthermore, there were no clear data about better efficiency of tandem transplant instead of single transplant 2012. Lenalidomide maintenance was not covered by health insurance at the time of diagnosis. We suggest, that patients with high risk cytogenetic aberrations, like CTH, could profit from lenalidomide maintenance therapy.

Taken together, we can conclude that rapid development and aggressive progression of the disease in our patient was underlined by presence of complex chromosomal aberrations including chromothripsis 18 and amplification in 16p together with causal mutations in several genes associated with hematologic malignancies. While most of found genetic changes have strong association with the development, course and prognosis of the disease, none of them except gain 1q21 are detectable by conventional FISH probe panel used in routine diagnosis. Based on the above-mentioned facts, implementation of genome-wide screening techniques, such as array-CGH, could improve the means of genetic diagnostics in MM and should be a part of genetic investigation in routine diagnostics in patients with MM.

## Additional files

Additional file 1: Table S1. Aberration list for CNAs detected by array-CGH in MM patient with chromothripsis 18. Output from Agilent Genomic Workbench 7.0.4.0. (XLSX 13 kb)

Additional file 2: Table S2. Annotation table of 338 called SNP variants from 62 leukemia-associated genes detected NGS Leukemia panel from Sistema Genómicos. (XLSX 91 kb)

## Abbreviations

CGH: comparative genomic hybridization
CNAs: copy number alterations
CTH: chromothripsis
CVD: cyclophosphamid-velcade-dexamethasone
FISH: fluorescence in situ hybridization
LOH: loss of heterozygosity
MGUS: monoclonal gammopathy of undetermined significance
MM: multiple myeloma
NGS: next generation sequencing
PBSCT: autologous peripheral blood stem cell transplant
PCs: plasma cells
PR: partial remission
RAD: revlimid-adryamicin-dexamethasone
